# iTriplet, a rule-based nucleic acid sequence motif finder

**DOI:** 10.1186/1748-7188-4-14

**Published:** 2009-10-29

**Authors:** Eric S Ho, Christopher D Jakubowski, Samuel I Gunderson

**Affiliations:** 1 Rutgers University, Department of Molecular Biology and Biochemistry, Nelson Laboratories, Room A322, 604 Allison Rd, Piscataway, NJ 08854, USA

## Abstract

**Background:**

With the advent of high throughput sequencing techniques, large amounts of sequencing data are readily available for analysis. Natural biological signals are intrinsically highly variable making their complete identification a computationally challenging problem. Many attempts in using statistical or combinatorial approaches have been made with great success in the past. However, identifying highly degenerate and long (>20 nucleotides) motifs still remains an unmet challenge as high degeneracy will diminish statistical significance of biological signals and increasing motif size will cause combinatorial explosion. In this report, we present a novel rule-based method that is focused on finding degenerate and long motifs. Our proposed method, named iTriplet, avoids costly enumeration present in existing combinatorial methods and is amenable to parallel processing.

**Results:**

We have conducted a comprehensive assessment on the performance and sensitivity-specificity of iTriplet in analyzing artificial and real biological sequences in various genomic regions. The results show that iTriplet is able to solve challenging cases. Furthermore we have confirmed the utility of iTriplet by showing it accurately predicts polyA-site-related motifs using a dual Luciferase reporter assay.

**Conclusion:**

iTriplet is a novel rule-based combinatorial or enumerative motif finding method that is able to process highly degenerate and long motifs that have resisted analysis by other methods. In addition, iTriplet is distinguished from other methods of the same family by its parallelizability, which allows it to leverage the power of today's readily available high-performance computing systems.

## Background

Here we present a rule-based method to identify degenerate and long motifs in nucleic acid sequences. The widely accepted sequence motif finding problem formulation proposed by Pevzner and Sze in [1] is adopted in this article. We call an oligomer of length *l*, an *l*mer. A motif model is denoted by <*l*, *d*>, where *l *is the length of the motif, and *d *is the maximum number of mutations allowed with respect to the motif. An instance of a motif is termed *d*-mutant. Two *d*-mutants of the same motif must not differ by more than 2*d *differences. We call two *l*mers neighbors if their difference is ≤ 2*d*. Given *n *sequences, each of length *L *(could be of variable length), the goal is to locate the set of *d*-mutants in each sequence from the sample where the largest difference between any pair of *d*-mutants in the set is ≤ 2*d*. In the following we will briefly summarize two major motif finding approaches, viz. statistical and combinatorial. Readers who are interested in a more comprehensive survey about motif finding can refer to [2,3].

Position weight matrix is often used as a statistical scoring system to identify biological signals from background. This technique implies that biological signals consist in part of conserved nucleotides that are critically important for their potency. As a result, motifs discovered by this approach tend to contain relatively invariant nucleotides at a few positions. Many transcription factor binding site prediction methods are developed based on this approach. Gibbs sampling and expectation maximization are typical techniques employed by MEME [4,5], AlignACE [6], BioProspector [7], MDScan [8] and MotifSampler [9]. The primary advantage of this approach is its speedy runtime and minimal memory consumption. However, statistical overrepresentation will vanish when the size of the motif to the number of mutations ratio decreases. One improvement of this approach is to incorporate phylogenetic information in background estimation. Well-known examples of this approach include FootPrinter [10] and PhyloGibbs [11]. However, such an approach is challenged by multiple substitutions occurring in distant species or motif searching in a single species. Some other methods train a Markov model to capture nucleotide dependency information of known binding sites in order to make prediction for unseen cases. One extension of the Markov model was reported in [12]. The authors incorporated several features, such as gaps and polyadic sequence elements, to handle diversified transcription factor binding sites.

An alternative to a statistical approach is the combinatorial or enumerative approach [1] where the observable biological signals are believed to be the variation of a hidden motif, and they do not exhibit conspicuous conservation at any particular position, and yet they are similar to each other. This approach is suitable for families of biological signals where the targeting proteins do not rely on a few conserved nucleotides at fixed positions. Instead the overall binding affinity is determined cooperatively by nucleotides in a region. Many such examples are found in precursor RNA processing signals including the pyrimidine-rich region near 3' splice sites and the U/GU-rich region downstream of polyadenylation sites. One fundamental problem faced by the enumerative approach is the exponential growth of computing resources when the size of the motif increases. To circumvent this, existing methods such as WINNOWER [1], MotifEnumerator [13], MITRA [14], TIERESIAS [15], Gemoda [16] and PMSprune [17], employ various elegant pruning strategies to abandon unpromising pursuits as early as possible.

Both enumerative and statistical approaches have proven to be valuable in analyzing real biological examples and both approaches are complementary to each other. In most situations when little prior knowledge is known about the motif, we believe both approaches should be considered. Since our focus is on solving degenerate and long motifs, we adopt the enumerative approach that is guaranteed to find the optimal motif by applying a novel rule-based algorithm to identify all optimal motif candidates without the expense of exploring the entire 4^l ^space exhaustively. In addition, our algorithm is designed to be highly parallelizable so as to exploit today's parallel computing technology in handling massive biological data. As a proof of concept, we will evaluate our algorithm using the simulated data described in [1]. Also we will show our method is able to identify motifs in real promoter sequences, and 5' and 3' untranslated regions (UTR) from different species. Results show that our method can solve highly degenerate and/or longer motifs that overwhelm the capabilities of other methods. Furthermore, we have compared the prediction accuracy of our method with the statistical motif finding methods mentioned above and find that our method is equal to and sometimes better than these methods. Besides *in-silico *simulations, we have also verified our prediction of downstream polyadenylation motifs for three human genes using a dual Luciferase assay. Our software is developed in C++ and standard template library (STL). It has been tested on Linux platform. Interested readers can download the software freely from this website http://www.rci.rutgers.edu/~gundersn/iTriplet.

## Methods

### iTriplet Algorithm

Our rule-based enumerative algorithm is named iTriplet. It stands for inter-sequence triplets. A triplet consists of three neighboring *l*mers (less than 2*d *differences from each other) sampled from three different sequences. The 'inter-sequence' part of the iTriplet algorithm systematically explores tripartite combinations of *l*mers from different sequences in order to identify motif(s) that span all sequences in the sample. The span of a motif refers to the number of sequences containing its *d*-mutant. For clarity, we will explain our method by limiting to only one motif in the sample, and every sequence contains at least one occurrence *d*-mutant of the motif even though our method can deal with multiple motifs and 10-20% of contamination. We will describe our iTriplet algorithm in two parts: the 'inter-sequence' part will be discussed first, followed by the Triplet algorithm.

### The inter-sequence part of iTriplet

If sufficient number of sequences are given and the motif model is not highly degenerate, i.e. small *d *with respect to *l*, the likelihood that an *l*-sized motif can span through all sequences by chance is rare. Based on this insight, we utilize the span of a motif as the indicator to identify unusual motifs in a sample.

The inter-sequence part of iTriplet consists of two stages: initialization stage and expansion-pruning stage. Below is the description of the procedure.

Given a set of *n *sequences and a motif model <*l*, *d*>, randomly designate two sequences from the sample as reference sequences, namely *R1 *and R2, and the rest as non reference sequences S_1_, S_2_, ..., S_n-2_.

**Figure 1:**
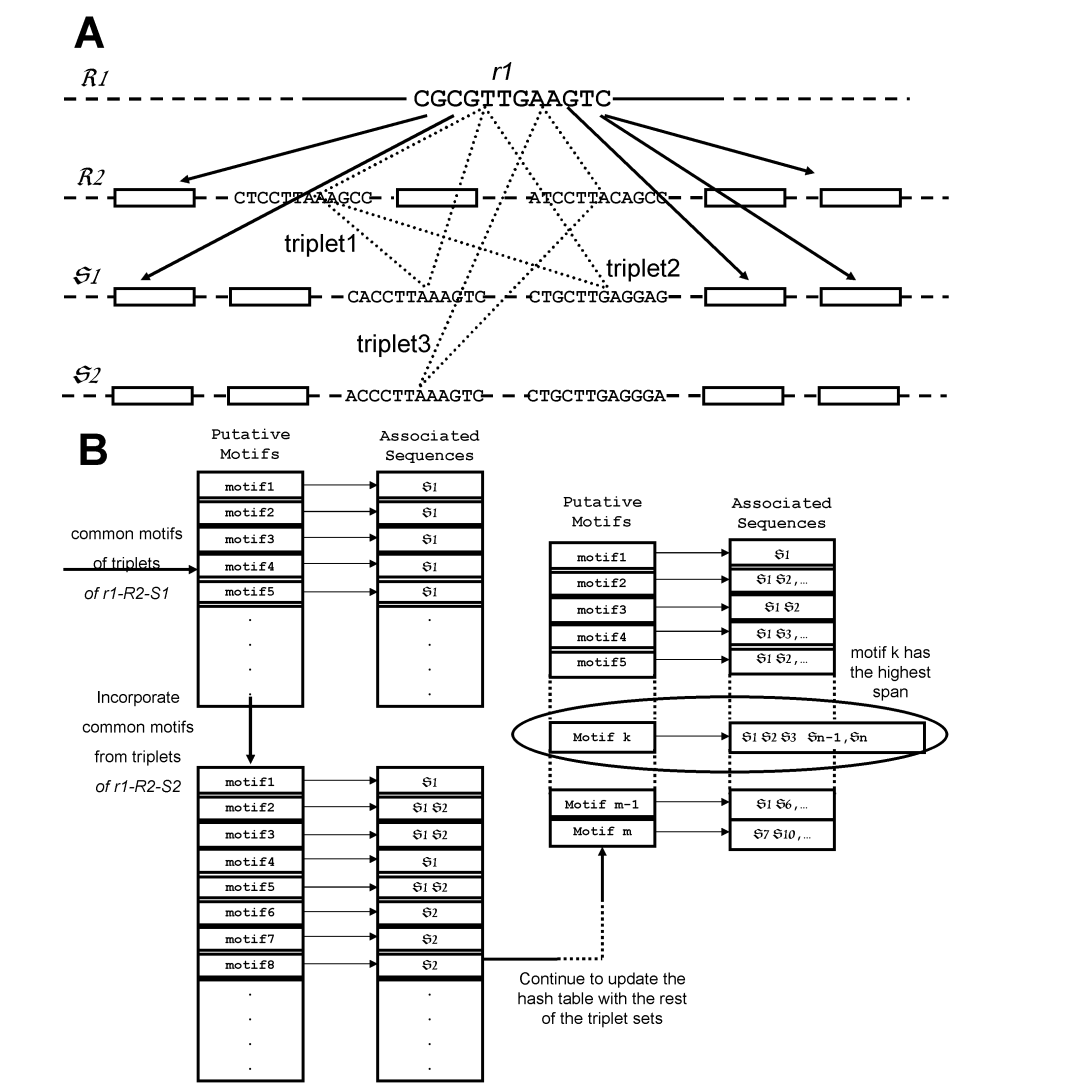
**Inter-sequence algorithm**. (A) For each *l*mer *r*1 in *R*1, identify 2*d*-mutants in sequences R2, S1, S2, ... The rectangular box represents the 2*d*-mutant of *r*1. The dotted line triangle represents a triplet. (B) Hash table to keep track of the span of the putative motif. Hash table consists of two parts viz. key and value. In this case, the key is the putative motif; value is a list of unique sequence IDs. Putative motifs are produced by the Triplet algorithm. They are common motifs to triplets.

Initialization stage: Randomly select an *l*mer (*r1*) from *R1 *and a non reference sequence, say S_i_. Identify all possible triplets based on *r1*, *l*mers from sequences *R2 *and S_i _as illustrated in Figure 1A. For each triplet, identify the set of motif(s), if any, common to the triplet using the Triplet algorithm (will be discussed later). Store the returned common motif(s) and its associated sequence IDs in a hash table as shown in Figure 1B.

Expansion-pruning stage: Randomly select an unprocessed non-reference sequence, say S_j_. Similar to the initialization stage, identify all triplets based on *r1*, *l*mers from sequences *R2 *and S_j_. Identify the set of common motifs of all triplets using Triplet algorithm and store them in the hash table. Prune the hash table by removing motifs with span not covering all processed sequences so far. If the hash table is not empty after pruning, repeat the expansion-pruning stage with the next unprocessed non-reference sequence. If the hash table is empty after pruning, return to the initialization stage, randomly pick a different *l*mer (*r1*) from *R1*, and repeat the same two-stage inter-sequence process again until all *l*mers in *R1 *have been processed. If all non-reference sequences have been processed and the hash table is not empty, then return motif(s) in the hash table to the calling program.

As described above, the processing of different *l*mer *r1 *in *R1 *are completely independent of each other. It means that they can be executed simultaneously wherein not even a single synchronization point is required. Therefore, given M processors, the algorithm can trigger up to (M-1) concurrent processes simultaneously. Theoretically, the performance gain by parallelizing this step is (M-1) times for a M-processor system where one processor is designated for overall coordination purposes. Our current parallel version of iTriplet is implemented based on this idea.

### The Triplet part of iTriplet

The purpose of this part of the algorithm is to uncover the complete set of motifs common to all members of the triplet in a deterministic and efficient way. The clues solely come from the similarities and differences among the three *l*mers rather than the enumeration of all possible *l*mers. It is efficient because the number of motifs shared among all three *l*mers should be small. By example, the estimated probability of any three *l*mers to share at least one common motif for models <12,3> and <30,9>, is 5.47 × 10^-4 ^and 2.97 × 10^-4^, respectively.

**Figure 2:**
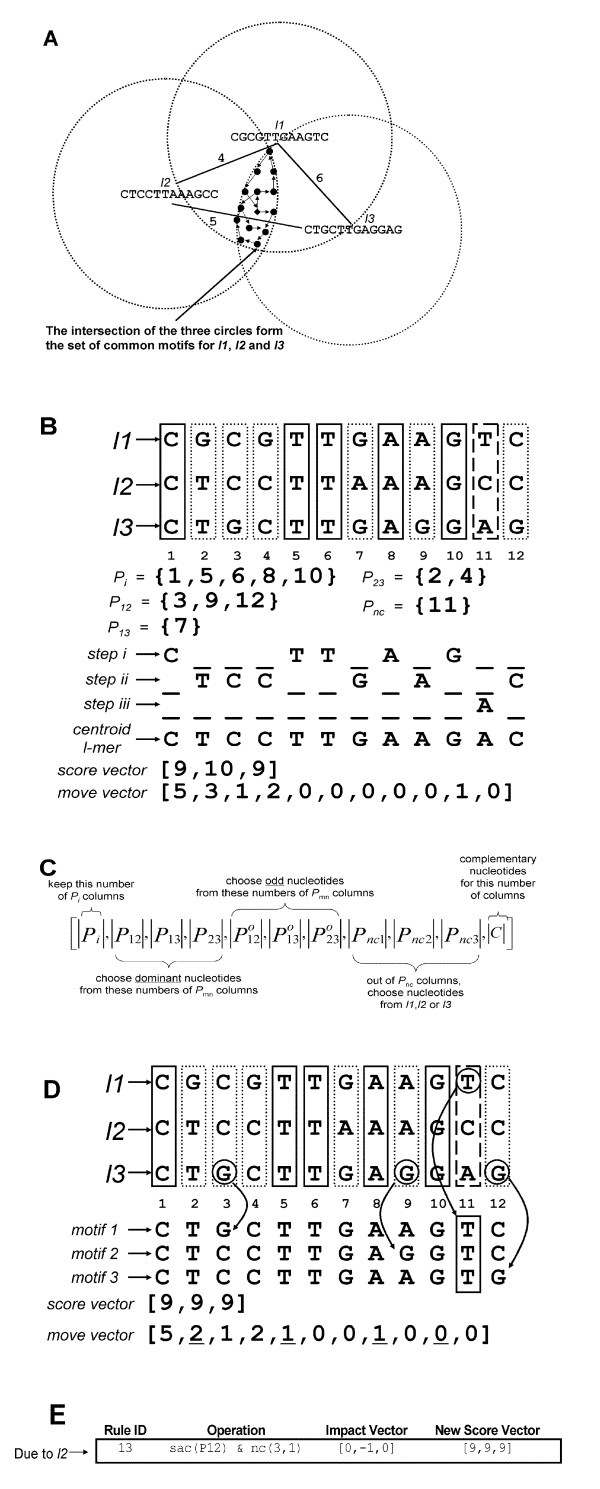
**Intuition of Triplet algorithm**. (A) Intuition of Triplet algorithm. A triplet consists of 12 mers *l*1, *l*2 and *l*3. *l*1 and *l*2, *l*1 and *l*3, and *l*2 and *l*3 contain 4, 6 and 5 differences respectively as labeled in the lines connecting them. Use the 12 mer as the center to draw an imaginary circle. Each circle denotes the set of neighboring 12 mers that are no more than 3 differences from the center 12 mer. In other words, each circle represents the set of putative motifs that generate the center 12 mer. Note that we do not actually generate the set of putative motifs. Centroid *l*mer is denoted by a diamond shape dot. The goal of the algorithm is to uncover all members of the set in the intersection (dark gray) of the three sets. (B) Centroid *l*mer construction. Shown are three patterns of columns viz. same nucleotide in three 12 mers *P*_i _(solid line vertical boxes in positions 1, 5, 6, 8 and 10), all different nucleotides across three 12 mers *P*_nc _(vertical box with dashed boundary in position 11), and two out of three 12 mers having the same nucleotides *P*_mn _(dotted line vertical boxes in positions 2, 3, 4, 7, 9, and 12). The centroid *l*mer is constructed in stage 1 of Triplet algorithm described in the text. The number of identical positions between the centroid *l*mer and *l*1, *l*2 and *l*3, is represented by the score vector and the selection of nucleotides encoded in move vector (C) Structure of move vector. (D) Exploratory scheme discovery from stage 2 of Triplet algorithm. Centroid *l*mer constructed in Figure 2B is modified by the composite operation of sac(P_12_) and nc(3,1) to create three extra motifs near its neighborhood. (E) Example of applying rule 13 to create a new move vector in (D).

Before we describe the algorithm, we need to define two main data structures used by this algorithm viz. move vector and score vector. The three *l*mers passed into this process are stacked up conceptually to form *l *numbers of three-nucleotide tall columns as shown in Figure 2B. These columns must fall into one of the three patterns: (I) with identical nucleotides denoted by *P*_i_; or (II) with all different nucleotides, denoted by *P*_nc_; or (III) with two out of three nucleotides being the same, denoted by *P*_mn _where m and n denote the indices of the two *l*mers with dominant nucleotide. We will show later that common motifs can be discovered by various ways of selecting nucleotide from these three types of columns. Such selection is captured in a move vector which is illustrated in Figure 2C. In addition, each move vector is associated with a score vector which is defined as [*i1*, *i2*, *i3*], where *i1*, *i2 *and *i3 *denote the numbers of identical positions between the motif represented by the move vector and the three given *l*mers *l1*, *l2 *and *l3*, respectively.

Triplet algorithm consists of three stages: 1) centroid *l*mer construction, 2) exploratory scheme discovery, and 3) motif generation. Below is the description:

Stage 1: centroid *l*mer construction. Given a triplet of three *l*mers from the calling program, identify the three column types *P*_i_, *P*_mn _and *P*_nc _as discussed above. Check if the triplet satisfies this inequality: *l*-*d *≤ |*P*_*i*_| + |*P*_*mn*_|*2/3+|*P*_*nc*_|*1/3 (for derivation, see Additional file 1) where |*P*_i_,|, |*P*_mn_| and |*P*_nc_| denote the number of *P*_i_, *P*_mn _and *P*_nc _patterns respectively. If the given triplet fails to satisfy this inequality, return no common motif and exit. Otherwise take these three steps to construct the initial move and score vectors: i) take the common nucleotides from columns *P*_i_, ii) take the dominant nucleotides from *P*_mn_, and iii) for columns *P*_nc_, take the nucleotides from the *l*mer which is currently farthest from the work-in-progress centroid *l*mer produced by the previous two steps. Pass the newly created move and score vectors to stage 2 for further processing.

Stage 2: exploratory scheme discovery. Based on the excess score(s) (> *l*-*d*) in one or more of the three values in the initial score vector, formulate alternative ways to select nucleotides from *P*_i_, *P*_mn _and *P*_nc _patterns through the 61 rules (will be discussed later). An execution of a rule produces a new set of move vector(s) and its associated score vector. Repeat stage 2 processing of the new move vector(s) until all newly generated score vector(s) becomes [*l-d, l-d, l-d*] i.e. no excess score. Pass all move and score vectors generated in this stage to stage 3.

Stage 3: motif generation. Generate motif by going through each value in the move vector, and select the specified number of column patterns and associated nucleotides accordingly. When all move vectors are processed, return all motifs to the calling program.

Regarding the rules mentioned in stage 2 of Triplet algorithm, they are actually made of five basic operations listed in Table 1. These five basic operations are the only possible alternatives to the selections which produce the centroid *l*mer. The basic operation can be applied individually or be combined with one other basic operation to act like a single operation, namely a composite operation. Basic or composite operations act on the current move vector in the light of its score vector. To facilitate searching, we pack the basic/composite operation and its impact or changes on the current score vector, namely impact vector, into a new construct called rule as shown in Figure 2E. These 61 rules are further organized into three non-mutually exclusive groups, each group has 42 rules, according to which *l*mer in the triplet possesses excess score (full list can be found in the Additional file 1). The decision to select a rule is determined by the three conditions. First, it has not been chosen already. Second, the three values of the new score vector, obtained by the addition of the impact vector and the current score vector, must be ≥ *l-d*. Third, the triplet contains the column pattern(s) required by the basic and/or composite operation. Notice that every rule will reduce the total score value of the new score vector. It means that successive applications of these rules will eventually create a score vector of its minimum score values [*l-d*, *l-d*, *l-d*] and that marks the terminal state.

**Table 1:** Five basic operations for triplet processing of iTriplet algorithm

Operations	Description	Examples based on Figure 2D if possible
sac(P_mn_)	Instead of choosing the dominant nucleotide from P_mn _column, choose the odd nucleotide.	sac(P_12_), take 'G' at position 3 from *l*3 instead of 'C' from *l*1 or *l*2
compl(P_mn_)	Instead of choosing the dominant or odd nucleotide from P_mn _column, choose nucleotides complementary to them.	Apply on the 2^nd ^column, compl(P_23_), take nucleotides complementary to 'G' and 'T', i.e. choose 'A' or 'C' for position 2.
nc(i, j)	Instead of taking nucleotide from *l*mer_i_, choose from *l*mer_j _in a P_nc _column.	Apply nc(3,1) to position 11. Instead of choose 'A' from *l*3, choose 'T' from *l*1 at position 11.
nc(i,0)	Instead of taking nucleotide from *l*mer_i_, choose from the complementary nucleotide of a P_nc _column.	Apply nc(3,0) to position 11. Instead of choose 'A' from *l*3, assign the complementary nucleotide 'G' to position 11.
sac_i(P_i_)	Instead of keeping the nucleotide identical to all lmers in the triplet, take the three complementary nucleotides.	Apply sac_i(P_i_) to position 1. Take 'A', 'G' or 'T' instead of 'C' at position 1.

Regarding stage 3, one move vector may generate more than one motif. For the example in Figure 2D, the new move vector due to rule 13 is [5,2,1,2,1,0,0,1,0,0,0]. The first value specifies to select the nucleotides from the five *P*_*i *_column patterns which are found in positions 1, 5, 6, 8 and 10 (see Figure 2B). Since there are exactly five *P*_*i *_column patterns, only one way is possible. The second value of the move vector specifies to choose dominant nucleotides from two *P*_12 _column patterns out of three and to choose the odd nucleotide from the remaining one. It will generate three possibilities. The rest of the values in the move vector will be processed similarly.

We have given the full description of iTriplet algorithm. Regarding the correctness of the algorithm, at this stage, we have not come up with a theoretical proof yet, however we have conducted extensive testing of more than 14,000 cases including models <11,2>, <12,3>, <13,3>, <15,4>, <28,8> and <40,12>; over 2,000 cases per model. In each case, we had generated 20 sequences each of length 600 with all nucleotides occurring equally likely. In each sequence, a single *l*-size *d*-mutant was planted at a random location. After each run, we checked whether the returned motif from iTriplet was the same as planted or not. iTriplet performed correctly for all cases.

### Time and Space Complexities of iTriplet

The inter-sequence part of iTriplet mainly iterates all combinations of triplets among sequences. Therefore, for model <*l, d*>, we estimate the time complexity of the inter-sequence part of iTriplet to be *O*(*nL*^3^*pl*) where *n*, *L *and *p *are the number of sequences, length of sequence and probability to form a triplet that shares at least one motif. As discussed before, we estimate *p *should be in the range of 10^-4^, and *L *should normally be 10^2^. Therefore, the effective time complexity of the inter-sequence part ranges from *O*(*nLl*) to *O*(*nL*^2^*l*). Stage 2 of Triplet part should generate all possible score vectors as long as the score value between each *l*mer and the centroid *l*mer is at least *l-d*. In the worst case scenario, there are *d*^3 ^score vectors. The generation of actual motifs based on the move vector in step 3 should depend on the size of the motif *l*. Therefore the time complexity of Triplet is *O*(*d*^3^*l*). Hence the overall time complexity of iTriplet is *O*(*nL*^3^*pl*^2^*d*^3^). For PMSprune, the time complexity is *O*(*nL*^2^*N(l, d)*), where *N(l, d) *is . After eliminating the common terms, the main difference lies in the growth of *Lpl*^2^*d*^3 ^and *N(l, d) *in iTriplet and PMSprune, respectively. When the motif model is small, *N(l, d) *is smaller than *Lpl*^2^*d*^3^. However, when *l *increases, the combinations of *N(l, d) *grows exponentially. iTriplet's space complexity depends on the degeneracy of the model, therefore it is *O*(*N(l, d)*) before pruning. After pruning, the space requirement will shrink.

## Results and Discussion

### Simulated data

In order to examine how iTriplet method can solve more degenerate and longer motifs, we compared it with some well known enumerative methods using simulated data. The simulated sequences were generated as described in the Additional Materials section. Simulated datasets were constructed using a wide range of *l *and *d *parameters in order to compare the performance of different methods in dealing with various sizes of the motif and/or noisy situations. The sequential version of our method was compared with three other well-known methods that have the same focus to guarantee finding the optimal motif viz. MotifEnumerator [13], PMSprune [17], and RISOTTO [18]. Sequential tests were conducted on a Linux machine equipped with an Intel P4 3 GHz processor and 2 Gbytes of memory. All methods can successfully identify the planted motifs in the simulated dataset unless the runtime was longer than 6 hours. We also repeated the same set of tests for the parallel version of iTriplet on a three-node Linux cluster equipped with the same processor as a sequential test. Results are tabulated in Table 2. The second column of Table 2 is the neighborhood probability of each model, which is the probability that any two *l*mers differ by no more than 2*d *by chance, a good indicator to reflect the degree of degeneracy of the model.

**Table 2:** Methods comparison on simulated datasets.

Models	Neighborhood Probability	MotifEnumerator	RISOTTO	PMSprune	iTriplet	iTriplet (parallel)
11,2	0.7%	6 s	2.2 s	1 s	2 s	1 s
12,3	5.4%	1 m	40 s	4 s	33 s	18 s
13,3	2.4%	2 m	33 s	2 s	6 s	4 s
14,4	11%	-^a^	8 m	1 m	3 m	2 m
15,4	5.6%	-	6 m	16 s	36 s	19 s
16,5	19%	-	82 m	13.5 m	26 m	13 m
18,6	28%	-	-^b^	-^b^	3 h	1.5 h
19,6	18%	-	-	-	27 m	14 m
24,8	23%	-	-	-	4 h	2 h
28,8	3%	-	-	-	19 s	10 s
30,9	5%	-	-	-	2.3 m	1.5 m
38,12	7%	-	-	-	1 h	33 m
40,12	3%	-	-	-	5 m	4 m

Neighborhood probability refers to the probability that two *l*mers differ by no more than 2*d *differences. The formula to calculate neighborhood probability is stated in the Additional file 1. Time is measured in seconds (s), minutes (m) or hours (h). (a) MotifEnumerator ran out of memory for *l *greater than 13. (b) Program took more than 6 hours to handle for the model <18,6> or longer. For the parallel version of iTriplet, reported runtime is the longest lapse time required for all nodes to finish.

For short motifs (<16 nucleotides) iTriplet is comparable to the fastest (PMSprune) and is significantly faster than MotifEnumerator and RISOTTO. When motif length is longer than 16, all other methods take longer than 6 hours to process. Note that iTriplet is able to process highly degenerate <18,6> and <24,8> models which cannot be handled by these other three methods as well as other statistical based methods such as MEME, MotifSampler and BioProspector. Based on these results, we learned that the performance of all methods depends on *l *and *d*, but to a different extent. Intriguingly, the runtime of PMSprune quadrupled, though still very fast, when *l *increased from 12 to 15 even though the neighborhood probability remained relatively at the same level. A similar trend is also observed in RISOTTO but with even higher fold increment in runtime. Such a phenomenon is not observed in our method. When neighborhood probability is doubled in models <12,3> versus <14,4>, and <14,4> versus <16,5>, the runtime of PMSprune increased 15 and 13.5 times respectively and RISOTTO increased 12 and 10 times respectively whereas iTriplet only increased 6 and 9 times, respectively. Based on these observations, we can understand that the algorithms employed by RISOTTO and PMSprune are quite sensitive to both *l *and *d *even when the neighboring probability remains at the same level. Thus RISOTTO and PMSprune take a longer time to search for the optimal motif; whereas the combined effect of *l *and *d *on performance was less severe for iTriplet. This explains why RISOTTO and PMSprune encountered difficulty in handling longer motif models. This does not exclude that iTriplet is unaffected by large *d *(high degeneracy). But one distinctive feature of our algorithm is that it can split the task into smaller subtasks which can be run independently in parallel. When comparing sequential and parallel versions of iTriplet, the parallel version averaged 1.77 times performance gain in a three-node cluster that is quite close to the theoretical gain 2.0. Testing based on the simulated data revealed that different methods have different tradeoffs in tackling the general <*l, d*> motif problem therefore further investigation is still needed to cope with various challenges of this problem.

### Real biological sequences

Besides simulated datasets, we tested our method using multiple sets of real biological sequences. One issue with real biological sequences is the lack of prior knowledge about the size and maximum numbers of mutations permitted by the motif. The optimal motif(s) comes from the model having the smallest neighborhood probability and produces the least number of motifs. In order to pin down the optimal motif, the algorithm must be run for a range of *l *and *d*. But we have found that the search of the optimal *l *and *d *can be done methodically by making use of the neighborhood probability of each model. In the situation when iTriplet has found too many motifs for the specified model then we can conclude that the model is too lax and so a more stringent model should be used, by increasing *l *or reducing *d *or both at the same time. Alternatively, once a satisfactory model is found, one can look for shorter models with similar neighborhood probability if the shorter alternative gives a similar result. In order to ease the effort for searching for the optimal model, iTriplet provides an autonomous mode option. Under autonomous mode, the program will explore various models using the strategy just described, and return the best models with motif length from 6 to 40 bases and maximum number of differences from 1 to 12. But the user also has the option to limit the size of motif to a specific range. Although many models are examined, only a very limited numbers of models, usually none or one, can provide the optimal motif unless the given sequences contain multiple motifs. Several reasons are that a slight change in the size and/or the maximum number of mutations will result in a substantial change in neighborhood probability which can be seen in Table 2. As mentioned in the Background section, we have included promoter and 5' UTR regions from four genes commonly chosen as test cases for motif finding algorithms [10,14,17]. In addition, we have also added a set of 3' UTR sequences in our test in order to understand how our method performs in other regions of a gene. Table 3 summarizes the prediction by iTriplet for various genes and genomic regions.

**Table 3:** iTriplet prediction using real biological sequences.

**Gene**:	Preproinsulin (IEB1) promoter+5' UTR		Remarks
iTriplet	GTYYGGAAAYTGCAGC**YTCAGCCCC**		<25,2> model
PMSprune	CAGC**CTCAGCCCC**TT		Ref. [17]
MITRA	C**CTCAGCCCCC**		Ref. [14]
Published	CTCAGCCCCCAGCCATCTGCCGACCCCCCC		Transfac ID: R04457

**Gene**:	**DHFR (promoter+5' UTR)**		**Remarks**

iTriplet	**RWSTSGCGCSAAAC**Y		<15,3> model
PMSprune	**ATTTCG**T**G**GGC**A**		Ref. [17]
MITRA	TGCA**ATTTC**GC**GCCA**AAC		Ref. [14]
Published	ATTTCGCGCCAAA		Transfac ID: R01928

**Gene**:	**Metallothionein promoter+5' UTR**		**Remarks**

iTriplet	TTT**TGCRC**T**CG**YCCC		<15,1> model
PMSprune	CTC**TGC**A**C**A**CGG**CCC		Ref. [17]
MITRA	**TGCGCCCGG**		Ref. [14]
Published	TGCGCCCGG		Transfac ID: R08298

**Gene**:	**c-fos serum response element promoter+5' UTR**		**Remarks**

iTriplet	**CCATATTAGGAC**ATCTGCGT		<20,1> model
PMSprune	**CCA**AAT**TT**G		Ref. [17]
MITRA	**CCATATTAGGACA**		Ref. [14]
Published	CAGGATGTCCATATTAGGACATC		Transfac ID: R00466

**3'UTR Regulatory Elements**	**iTriplet Prediction only**	**Published**	**Remarks**

AU-rich (ARE)	**TTTTATTTATTTT**T	WWTTATTTATTWW	<14,3> model
Cytoplasmic Polyadenylation element (CPE)	**TTTTAAT**	TTTTAT and TTTTAAT	<6,1> model
Pumillio binding element (PBE)	**T**K**T**W**AATA**	TGTAAATA	<8,1> model

Motif predicted by iTriplet is presented in consensus sequence. Bold and underlined sequence represents correctly predicted nucleotide. Transfac IDs are obtained from TRANSFAC database [37]

Multiple motifs are often identified by iTriplet for real biological sequences. Four reasons account for this: 1) the number of sequences considered is small, mostly 4 in our test therefore resulting in a higher chance to encounter random span, 2) a naturally occurring recognition site is not necessarily represented by one consensus, 3) it is possible for the biological sequence to carry more than one signal especially in the 3' UTR, and 4) the presence of low complexity repeats.

Therefore we need a scoring system to filter out random from genuine motifs. Since only a small number of sequences are given, the set of true motif instances must resemble each other more than a set of random *l*mers; otherwise no conclusion can be made. As we have discussed in the inter-sequence algorithm section, if members of the triplet are very similar to each other, the intersection will become big, i.e. high numbers of common motifs. Based on this property, we derived a straightforward scoring system based on the numbers of common motifs uncovered to support whether the finding is statistically significant. Due to this, the 5' and 3' overlapping neighbors of the true motif are often included as part of the prediction as well. Therefore in some cases of the genes listed in Table 3, the predicted motif is longer than the model specified. Each prediction is a consensus of a number of common motifs. The method of constructing the consensus is similar to the frequency plot of Weblogo [19]. Nucleotides with frequency at a position greater than 30% will be included in the consensus sequence. As can be seen from Table 3, our predictions for promoter and 5' UTR sequences, and 3' UTR regulatory elements are largely consistent with published experimental data.

### Sensitivity and specificity test

We also measured the prediction accuracy of iTriplet in predicting transcription factor binding sites in E. Coli. These binding sites are experimentally validated and documented in the RegulonDB database [20]. The test was conducted using the three-level testing framework described in [21]. Under this testing framework, the prediction made by a method is measured at the nucleotide, binding site and motif levels. In the first and second levels, i.e. nucleotide and binding site levels, sensitivity, specificity, performance coefficient and *F*-measure are computed based on the true positive (*TP*), false positive (*FP*) and false negative (*FN*) information gathered by comparing the predicted and actual binding sites. Performance coefficient and *F*-measure were originally proposed by [1,22] and [21] respectively. Both of them have the advantage to combine sensitivity as well as specificity perspectives into a single number so as to ease interpretation. The formula for these four measurements can be found in the Additional Materials section. Note that at the binding site level, a prediction is considered correct when the predicted binding site overlaps with the actual binding site by at least one nucleotide. These four measurements were calculated for each transcription factor individually. Averaged measurements of all transcription factors are used for method comparison. The Kihara group [21] also suggested a third level assessment that is motif level. The rationale of this extra level test is to assess the adaptability of the method to make correct predictions for a wide range of transcription factors. The motif level measures the fraction of correct predictions out of all binding sequences and transcription factors. iTriplet was compared with the top three performers, i.e. MEME, BioProspector and MotifSampler, listed in Table 1 of [21], and WEEDER [23]. For each method, the parameter setup was adopted from [21] except that no background sequence information was used for BioProspector. Motif length was set to 15, the same length used in [21] except WEEDER where the maximum supported length is 12. We chose the maximum differences in the range from 3 to 5. For accuracy measurements, the top five predictions were used for the three selected methods. But in our case, we selected only the highest score consensus motif(s) instead of the top five used in [21]. Although only BioProspector and MotifSampler exhibit variation in prediction even for the same input sequences, in order to maintain fair treatment, we still repeat the test ten times for all methods. Table 4 shows the averaged measurements of iTriplet together with four other motif finding methods. iTriplet has demonstrated better prediction accuracy than the other four methods at both nucleotide as well as binding site levels except the *F*-measure is second at the nucleotide level. However, our *mSr *and *sSr *scores are ranked third mainly because these two measurements tend to favor methods with high sensitivity regardless of specificity. In the extreme situation, if a method predicts all nucleotides are part of a motif, it will score 1 for *mSr *and *sSr*. This point is further evidenced by the disproportionality of sensitivity and specificity of the other three methods except WEEDER at both nucleotide and motif levels. Therefore we think *PC *and *F*-measure are fairer measurements of prediction accuracy than *mSr *and *sSr*.

**Table 4:** Prediction accuracy of iTriplet versus four others motif finding methods.

Algorithms	Nucleotide	level			Binding	level			Motif	level
	*nPC*	*nSn*	*nSp*	*nF*	*sPC*	*sSn*	*sSp*	*sF*	*mSr*	*sSr*
iTriplet	0.195	0.292	0.322	0.286	0.319	0.489	0.418	0.422	0.853	0.591
MEME	0.180	0.551	0.214	0.296	0.258	0.733	0.280	0.397	1.000	0.817
WEEDER	0.128	0.274	0.245	0.208	0.263	0.538	0.332	0.367	0.833	0.532
BioProspector	0.102	0.372	0.129	0.179	0.212	0.704	0.224	0.328	0.986	0.670
MotifSampler	0.052	0.257	0.068	0.091	0.106	0.422	0.111	0.162	0.461	0.392

*PC*, *Sn*, *Sp *and *F *are performance coefficient, sensitivity, specificity and *F*-measure level respectively. Prefixes 'n' and 's' represent nucleotide or binding site level measurements respectively. *mSr *and *sSr *are motif and sequence level accuracy respectively.

### In vitro verification of predicted polyA downstream elements

**Figure 3:**
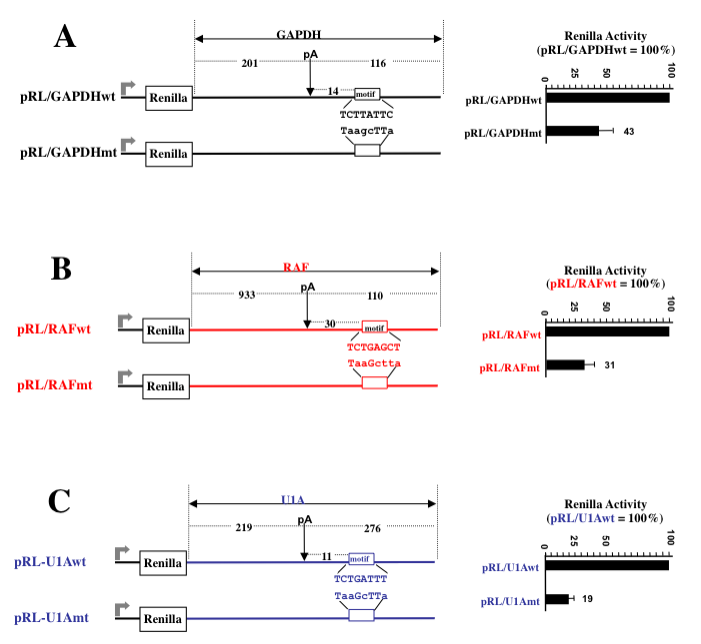
**Confirmation of predicted polyA downstream elements by dual Luciferase reporter system**. (A) pRL-GAPDHwt was made from a standard pRL-SV40 Renilla expression plasmid by replacing the SV40-derived 3'UTR and polyA signal sequences with the human GAPDH 3'UTR (NM_002046) and 116 nt past the PAS. pRL-GAPDHmt matches pRL-GAPDHwt but having Motif A mutated as shown. Plasmids were transfected into HeLa cells and Luciferase activity measured 24 hours later. Values for Renilla Luciferase were normalized to those obtained from a co-transfected Firefly Luciferase plasmid. The pRL-GAPDHwt plasmid expresses 2.2 fold more Renilla than pRL-GAPDHmt plasmid thus Motif A is enhancing expression by 2.2 fold. (B) pRL-RAFwt (NM_002880) was made like pRL-GAPDHwt but from the human RAF gene sequences as indicated. pRL-RAFmt matches pRL-RAFwt but having Motif A mutated as shown. These plasmids were transfected and analyzed as in panel A. (C) pRL-U1Awt (NM_004596) was made like pRL-GAPDHwt but from the human U1A gene sequences as indicated. pRL-U1Amt matches pRL-U1Awt but having Motif A mutated as shown. These plasmids were transfected and analyzed as in panel A.

To examine whether motifs predicted by iTriplet had biological activity, we chose to examine sequences important in the 3' end processing of mammalian pre-mRNA, in particular sequences found just downstream of the cleavage and polyadenylation site. Almost all eukaryotic mRNAs contain a post-transcriptionally-added polyA tail that is important for many aspects of mRNA function. According to one bioinformatic study, 54% and 32% of genes in human and mouse, respectively, contain more than one polyadenylation site [24]. The polyA tail is added at the polyA site (PAS) in the nucleus in a 2 two-step reaction consisting of a large cleavage complex that cleaves the pre-mRNA into two fragments followed by polyA tail addition to the upstream fragment [25]. Two main sequence motifs are important for cleavage/polyadenylation of mammalian mRNAs. The highly conserved and well-understood AAUAAA motif (called the polyA signal) is found 10-25 nt upstream of the PAS. The second motif is found 10-30 nts downstream of the PAS but is poorly understood due to its low conservation both in sequence and position. Although current bioinformatic approaches support the view that this motif is U/GU-rich [26], they provide only a limited understanding of what motif(s) lies in this downstream region. First, the exact identity of this putative downstream motif for a given mammalian gene is often ambiguous and indeed it is a distinct possibility that there will be multiple motifs including auxiliary motifs. Second, in some cases where the predicted motif was examined by an extensive mutational analysis, the data supported the existence of additional motifs important for polyA site function [27]. Thus the prediction of this downstream motif represents a type of problem suitable for analysis by iTriplet. To this end the downstream sequences of a set of genes was analyzed by iTriplet with the predicted motifs being indicated in Figure 3. According to a NMR structural study of the U/GU-rich binding protein CstF-64 [28], we believe the binding site should not be longer than eight nucleotides. Hence we applied a series of models ranging from 6 to 8 nucleotides long to nine genes of interest to us viz. U1A, SPR40, CDC7, DATF, LBP1, GAPDH, RAF, Mark1 and SmE. Results showed that model <8,2> yielded the best fit with the consensus TCTGATTT and this motif agrees with previous analysis performed by the Graber lab [26] that the downstream region consists of a transition from UG-rich to U-rich in the 5' to 3' direction. MEME [4,5] was used to process the same set of sequences with the resulting motif being BTRDGSCWSA that lacks such a transition.

To test whether the TCTGATTT motifs identified by iTriplet were functional, the dual Luciferase reporter system was used where Renilla Luciferase mRNA contained the entire 3'UTR plus sequences past the PAS of the gene of interest. A co-transfected Firefly Luciferase reporter was included that serves as an internal normalization control. As diagrammed in Figure 3, the plasmid pRL-GAPDHwt was made from a standard pRL-SV40 Renilla expression plasmid by replacing the SV40-derived 3'UTR and downstream polyA signal sequences with the human GAPDH 3'UTR and polyA signal region (NM_002046) including 116 nt past the polyA site. iTriplet predicted that GAPDH has a motif we call GAPDH Motif A that would potentially be important for polyA site activity. To determine if GAPDH Motif A is functional, we mutated it as shown to make plasmid pRL-GAPDHmt. Plasmids were transfected into HeLa cells and Luciferase activity was measured; values for Renilla Luciferase were normalized to those obtained from the co-transfected Firefly Luciferase control plasmid. The pRL-GAPDHmt plasmid expresses 43% less Renilla Luciferase than pRL-GAPDHwt, indicating Motif A enhances Renilla Luciferase expression by about 2.2-fold.

The same analysis was done in panels B and C but for the human RAF and human U1A genes, respectively. As can be seen the RAF Motif A enhances expression 3.2 fold and the U1A Motif A enhances expression by 5.1 fold. Here we have demonstrated the predictive power of iTriplet for these three genes however we do not exclude the existence of other binding sites that can also affect polyA activity of these genes.

## Conclusion

We have presented a novel rule-based algorithm called iTriplet to solve the challenging degenerate and long motif finding problem that was unsolved before. In addition, we have confirmed our prediction for real biological signals experimentally. The runtime of iTriplet is comparable to other well-known methods of the same design philosophy and is significantly better at analyzing longer motifs (>16 nucleotides). To our knowledge, iTriplet is the most parallelizable motif finding method in the family of guaranteed optimal motif finding algorithms developed so far. Furthermore we have shown that our method is very competitive in prediction accuracy when compared with other popular motif finding methods. Overall, our method has the superiority like other exact optimal motif finding methods to find the optimal motif in the absence of statistical overrepresentation and yet without sacrificing prediction accuracy. That said, no single method or approach is able to solve the general <*l, d*> motif problem completely in terms of guaranteed solution, speed, memory consumption and prediction accuracy. Thus, further research effort is needed to overcome various hurdles of this problem.

## Additional Materials

### Simulation data

We generated multiple sets of simulated sequences according to the <*l, d*> motif model formulated by Pevzner and Sze [1]. Each dataset consists of 20 sequences, each 600 nucleotides long. All nucleotides occur equally likely. In each sequence, a single *l*-size *d*-mutant is planted at a random location. We have prepared datasets for a wide range of <*l, d*> motif models, i.e. <11,2>, <12,3>, <13,3>, <14,4>, <15,4>, <16,5>, <18,6>, <19,6>, <24,8>, <28,8>, <30,9>, <38,12>and <40,12>.

### Untranslated region sequence data

In addition to simulated data, we also prepared and tested several sets of real biological data that can be split into two groups, one 5' upstream of the start codon, i.e. 5' UTR and promoter; and the other from the 3' UTR. For the 5' UTR-promoter group, we chose four genes that are commonly tested in other motif finding algorithms [10,14,17], namely, preproinsulin, DHFR, metallothionine, and c-fos. Homologous regions from four species were included for analysis using the Homologene database http://www.ncbi.nlm.nih.gov/sites/entrez?db=homologene from NCBI. To obtain the upstream promoter region, BLAT [29] was used to map the cDNA to the species genome provided by Genome browser [30]. Based on the 5' starting point of the cDNA, we then extracted promoter sequence from the genome. Further details of this sequence set are found in the Additional file 1.

Another set of real biological sequences is taken from the 3' UTR where AU-rich elements (AREs), cytoplasmic polyadenylation elements (CPEs), and Pumillio binding elements (PBEs) [31] were chosen. The AREs were derived from 30 experimentally validated human and mouse 3' UTRs [32]. These genes were also confirmed by the ARE database ARED 2.0 http://brp.kfshrc.edu.sa/ARED/[33]. Based on the accession numbers provided by ARED, we retrieved the cDNA sequences from NCBI's RefSeq database [34]. The 5' end of the 3'UTR begins right after the stop codon, However, the 3' end of the 3' UTR is not obvious because we have found that most of the cDNA sequences deposited in RefSeq database lack a poly(A) tail. In order to accurately determine the 3' end of the 3' UTR, we utilized expressed sequence tag (EST) data from the UCSC Genome Browser [35]. We first mapped each cDNA to the genome using BLAT. The true end of the 3' UTR should coincide with the endpoint of the EST. The more ESTs that end at the same spot as the cDNA, the higher confidence we have about the true end of the 3' UTR. The set of sequences we obtained are variable in length ranging from 92 to 1608 bases bringing the total sequence space to 23,022 bases. For CPEs and PBEs, we have used the five cyclin genes, B1, B2, B3, B4, and B5 from *Xenopus laevis *[31]. Complete annotation of the sequences can be found in the Additional file 1.

### Run-time performance

We compared the performance of our method with three other methods with the same enumerative design philosophy, viz. MotifEumerator, RISOTTO and PMSprune. Source codes were downloaded from these sites, MotifEnumerator from http://faculty.cs.tamu.edu/shsze/motifenumerator/, RISOTTO from http://kdbio.inesc-id.pt/~asmc/pub/software/RISO/riso-me-src.zip, and PMSprune from http://www.engr.uconn.edu/~jid02003/Jaime/pmsprune.c. They were compiled in the Linux ×86 platform according to the instructions documented in the respective websites.

### Transfection and Luciferase assays

Cell culture and transfections were done as previously described in [36]. For Luciferase assays, the cells were harvested after 24 hours and Luciferase measured using the Promega dual Luciferase kit (Promega, Madison, WI) measured on a Turner BioSystems Luminometer (Turner BioSystems, Sunnyvale, CA).

### Sensitivity and specificity test

For the sensitivity and specificity test, we adopted the three-level (nucleotide, binding site, and motif) testing framework proposed by the Kihara group [21]. Two sets of data, ECRDB70 and ECRDB62A, were downloaded from their website http://dragon.bio.purdue.edu/pmotif. These data were originally derived from the RegulonDB database [20]. The ECRDB62A dataset comprises 713 intergenic sequences containing binding sites for 62 transcription factors in E. Coli K-12. We filtered out duplicated sequences, transformed reverse strands into forward direction, and dropped transcription factors with less than three binding sequences. The final reconstructed dataset contains 379 distinct sequences from 36 transcription factors. At the nucleotide and binding site level, four different assessments were performed. Sensitivity (*Sn*) is defined as , where *TP*, *FN *stands for true positive and false negative respectively. Specificity (*Sp*) is defined as , where *FP *is false positive. We followed two other assessments that were described in [21] to combine *Sn *and *Sp*. Performance coefficient (*PC*) is defined as , which was originally proposed in [1,22]. The last assessment is called *F*-measure (*F*), which tends to penalize the imbalance of *Sn *and *Sp*. *F *is defined as . Both *PC *and *F *fall into the range of [0,1], with value 1 indicating perfect prediction. In addition to the nucleotide and binding site levels, the Kihara group proposed two other accuracy measurements viz. sequence accuracy (*sSr*) and motif accuracy (*mSr*). *sSr *is defined as  where *Ns *is the number of sequences having their motifs correctly predicted, and *N *is the total number of binding sequences of a transcription factor. The overall *sSr *is the average *sSr *of all transcription factors. *mSr *is defined as , where *Np *is the number of transcription factors with at least one correctly predicted binding site in the binding sequence set and *M *is the total number of transcription factors in the dataset. We compared our method with WEEDER [23] and the top three best-performing methods previously evaluated in [21]: MEME, BioProspector and MotifSampler. MEME, BioProspector, MotifSampler and WEEDER were download from http://meme.nbcr.net/downloads/, http://motif.stanford.edu/distributions/bioprospector/, http://homes.esat.kuleuven.be/~thijs/download/linux_3.2/MotifSampler and http://159.149.109.9/modtools/downloads/weeder1.3.1.tar.gz respectively. For WEEDER, we specified the organism to be E. Coli K12 "BEC" and the type of analysis "large".

### Availability and Requirements

Source code of iTriplet is freely available and requires no license requirement. The current version of iTriplet is only available on RedHat linux platform. It requires GNU g++ compiler version 3.4.4 or above and Python version 2.5.1 or above. Interested readers can download the software from our website via the following link http://www.rci.rutgers.edu/~gundersn/iTriplet. Detailed information about how to build and run the software, description of parameters and output can be found in the captioned website.

## Competing interests

The authors declare that they have no competing interests.

## Authors' contributions

ESH and SIG conceived and wrote the manuscript. CDJ conducted the Luciferase assays. ESH designed and developed the algorithm.

## Supplementary Material

Additional file 1 **iTriplet: a rule-based nucleic acid sequence motif finder**. Materials about promoter and 5' UTR sequences, 3' UTR sequences, probability of motifs, 61 rules to discover neighboring motifs, parallelization configuration, and help text of iTriplet. Click here for file
