# Supplementary material for: iTriplet, a rule-based nucleic acid sequence motif finder
Source: Algorithms Mol Biol. 2009 Oct 29;4:14. doi: 10.1186/1748-7188-4-14 (PMC2784457; doi:10.1186/1748-7188-4-14)
Supplement: Additional file 1 — iTriplet: a rule-based nucleic acid sequence motif finder. Materials about promoter and 5' UTR sequences, 3' UTR sequences, probability of motifs, 61 rules to discover neighboring motifs, parallelization configuration, and help text of iTriplet. [file 1748-7188-4-14-S1.doc]

Additional File 1: Supplemental Data

iTriplet : a rule-based nucleic acid sequence motif finder

Eric S. Ho, Christopher D. Jakubowski, and Samuel I. Gunderson*

Rutgers University, Department of Molecular Biology and Biochemistry, Nelson Laboratories, Room A322, 604 Allison Rd, Piscataway, NJ 08854, USA

Promoter and 5’ UTR Sequences

Preproinsulin (INS):

| Species | Accession No. | Length |
| --- | --- | --- |
| Human | NM_000207 | 500 |
| Chimp | NM_001008996 | 500 |
| Mouse | NM_008378 | 500 |
| Rat | NM_019130 | 500 |

DHFR:

| Species | Accession No. | Length |
| --- | --- | --- |
| Human | NM_000791 | 200 |
| Drosophila | NM_001043255 | 200 |
| Mouse | NM_010049 | 200 |
| Hamster | M13129 | 200 |

Metallothionein (MT2A):

| Species | Accession No. | Length |
| --- | --- | --- |
| Human | NM_005953 | 648 |
| Bovine | XM_586929 | 923 |
| Mouse | NM_008630 | 820 |
| Chimp | XM_526603 | 1065 |

c-fos

| Species | Accession No. | Length |
| --- | --- | --- |
| Human | NM_005252 | 755 |
| Bovine | NM_182786 | 739 |
| Mouse | NM_010234 | 745 |
| Rat | NM_022197 | 752 |
| Dog | XM_547914 | 755 |

Transfac IDs listed in Table 3 are obtained from TRANSFAC database 7.0 – public (<http://www.gene-regulation.com/cgi-bin/pub/databases/transfac/search.cgi>)

3’ UTR sequences

ARE sequences:

| Genes | Accession No. | Length |
| --- | --- | --- |
| c-fos human | NM_005252 | 775 |
| c-jun human | NM_002228 | 1277 |
| junB mouse | NM_008416 | 446 |
| c-myc human | NM_002467 | 463 |
| krox20 mouse (EGR2) | NM_010118 | 1188 |
| nur77 mouse (NR4A1) | NM_010444 | 528 |
| zif268 mouse (ERG1) | NM_007913 | 1167 |
| GM-CSF mouse | NM_009969 | 327 |
| IL-3 mouse | NM_010556 | 477 |
| IFN-beta human | NM_002176 | 195 |
| IL-11 human | NM_000641 | 1608 |
| c-myb human | NM_005375 | 1195 |
| Mda-7 IL-24 human | NM_006850 | 1083 |
| CD69 human | NM_001781 | 998 |
| CHOP/GADD153 DDIT3 human | NM_004083 | 224 |
| pim-1 human | NM_002648 | 1340 |
| IL-8 human | NM_000584 | 1255 |
| IL-6 human | NM_000600 | 427 |
| IL-10 human | NM_000572 | 1036 |
| IL-2 human | NM_000586 | 285 |
| IL-4 human | NM_000589 | 92 |
| MYCN human | NM_005378 | 913 |
| IL-1 beta human | NM_000576 | 604 |
| TNF alpha human | NM_000594 | 801 |
| PLAU plasminogen activator, urokinase human | NM_002658 | 939 |
| PLAUR Urokinase type plasminogen receptor human | NM_002659 | 313 |
| PAI-2 human | NM_002575 | 584 |
| EDN2 human | NM_001956 | 637 |
| glut1 human | NM_006516 | 1173 |
| CSF3 G-CSF mouse | NM_009971 | 672 |

Cytoplasmic Polyadenylation Elements (CPE) and Pumillio Binding Elements (PBE):

We have extracted 3’ UTR from these five genes from *Xenopus Laevis*:

| Genes | Accession No. | Length |
| --- | --- | --- |
| Cyclin B1 | J03166 | 108 |
| Cyclin B2 | J03167 | 175 |
| Cyclin B3 | AJ304990 | 203 |
| Cyclin B4 | AJ304491 | 80 |
| Cyclin B5 | AJ304992 | 151 |

Total number of motif instances

In a (*l*,*d*) model, motif is *l* nucleotides long and the model allows up to *d* point mutations at random positions out of the *l* nucleotides. For example, a (12,3) model has a 12nt motif and a motif instance carrying up to 3 point mutations from the motif. The total number of possible motif instances from a (12,3) model can be determined by the following formula:

In a (12,3) model, the total possible motif instances is 6,571.

Probability of a motif to encounter a motif instance

In a (12,3) model, the probability is 3.9167x10^-4^.

Probability that two *l*mers differ by less than 2d differences (neighborhood probability)

In a (12,3) model, the probability is 0.0544.

Expected maximum span of a motif

We assume all nucleotides occur equally. An estimation of the expected maximum span **s** of a motif is given by minimum s that satisfies, where L=length of sequence, *l*=size of motif, *p* = probability of encountering an *l*mer with ≤ d differences, and s is the number of sequences span by chance. (L-*l*+1)*p* is the estimated proportion of all possible *l*mers that will encounter an *l*mer with ≤ d differences in a sequence. It is an estimate because *l*mers in a sequence overlap with each other.

For example, in a (12,3) model, length of sequence is 600, *p* = 3.9167x10^-4^, if you consider all possible 12-mers, it is estimated that there is one 12-mer that can span 12 sequences by chance. Span by chance is directly related to *p*. Large *p* indicates a highly degenerate model, we expect random span to increase by this formula.

Inequality to check if *l*mers in the triplet share at least one common motif

Minimum numbers of identical positions between each *l*mer in the triplet and the common motif is (*l*-*d*), *l* = length of motif, *d* = maximum number of mutations.

Let’s denote the number of *P_i_*, *P_mn_* and *P_nc_* patterns by |*P_i_*,|, |*P_mn_*| and |*P_nc_*| respectively.

For *l*mer1, the number of identical positions must satisfy this:

*l*-*d* ≤ |*P*_i_,| + |*P*_12_| + |*P*_13_| + |*P*_nc assign to_ *_l_*_mer1_|

Similarly, for *l*mer2 and *l*mer3, it will be:

*l*-*d* ≤ |*P*_i_,| + |*P*_12_| + |*P*_23_| + |*P*_nc assign to_ *_l_*_mer2_|

*l*-*d* ≤ |*P*_i_,| + |*P*_13_| + |*P*_23_| + |*P*_nc assign to_ *_l_*_mer3_|

These three inequalities must hold simultaneously, so we summarize them together into one inequality:

3(*l*-*d*) ≤ 3|*P*_i_,| + 2|*P*_12_| + 2|*P*_13_| + 2|*P*_23_| + |*P*_nc assign to_ *_l_*_mer1_| + |*P*_nc assign to_ *_l_*_mer2_| + |*P*_nc assign to_ *_l_*_mer3_|

Since|*P*_mn_| = |*P*_12_| + |*P*_13_| + |*P*_23_|, and |*P*_nc_| = |*P*_nc assign to_ *_l_*_mer1_| + |*P*_nc assign to_ *_l_*_mer2_| + |*P*_nc assign to_ *_l_*_mer3_|, we can simplify the above inequality through these two substitutions and divide both sides by 3. Hence, the precondition for a triplet to share at least one common motif is:

Number of references when not every sequence contains a motif instance (contamination)

Let the percentage of sequences with a motif be *p*, and the total number of sequences is *n*. [(1-*p*)**n* + 2] numbers of sequences will be chosen as reference sequences. The iTriplet will then iterate all possible selections of two out of the reference sequences as *R1* and *R2* mentioned in the main text. Therefore by doing this, we can convert the problem to what is discussed in the main text.

61 Rules to discover neighboring motifs

Note: Rule IDs are not in consecutive order. For the description of operations, refer to Table 1 in the main text.

| Rule ID | Operation | Impact on Score Vector |
| --- | --- | --- |
| 1 | Sac(P_12_) | [-1,-1,+1] |
| 2 | Compl(P_12_) | [-1,-1,0] |
| 3 | Sac_sac(P_12_, P_13_) | [-2,0,0] |
| 4 | sac_compl(P_12_, P_13_) | [-2,-1,0] |
| 5 | Sac_sac(P_12_, P_23_) | [0,-2,0] |
| 6 | sac_compl(P_12_, P_23_) | [-1,-2,0] |
| 7 | Sac_nc(P_12_, (1,2)) | [-2,0,1] |
| 8 | Sac_nc(P_12_, (1,3)) | [-2,-1,2] |
| 9 | Sac_nc(P_12_, (1,0)) | [-2,-1,1] |
| 10 | Sac_nc(P_12_, (2,1)) | [0,-2,1] |
| 11 | Sac_nc(P_12_, (2,3)) | [-1,-2,2] |
| 12 | Sac_nc(P_12_, (2,0)) | [-1,-2,1] |
| 13 | Sac_nc(P_12_, (3,1)) | [0,-1,0] |
| 14 | Sac_nc(P_12_, (3,2)) | [-1,0,0] |
| 15 | Sac_nc(P_12_, (3,0)) | [-1,-1,0] |
| 81 | Nc(1,0) | [-1,0,0] |
| 84 | Nc(1,2) | [-1,1,0] |
| 85 | Nc(1,3) | [-1,0,1] |
|  |  |  |
| 24 | Sac(P_13_) | [-1,1,-1] |
| 25 | Compl(P_13_) | [-1,0,-1] |
| 27 | sac_compl(P_13_, P_12_) | [-2,0,-1] |
| 28 | Sac_sac(P_13_, P_23_) | [0,0,-2] |
| 29 | sac_compl(P_13_, P_23_) | [-1,0,-2] |
| 30 | Sac_nc(P_13_, (1,2)) | [-2,2,-1] |
| 31 | Sac_nc(P_13_, (1,3)) | [-2,1,0] |
| 32 | Sac_nc(P_13_, (1,0)) | [-2,1,-1] |
| 33 | Sac_nc(P_13_, (2,1)) | [0,0,-1] |
| 34 | Sac_nc(P_13_, (2,3)) | [-1,0,0] |
| 35 | Sac_nc(P_13_, (2,0)) | [-1,0,-1] |
| 36 | Sac_nc(P_13_, (3,1)) | [0,1,-2] |
| 37 | Sac_nc(P_13_, (3,2)) | [-1,2,-2] |
| 38 | Sac_nc(P_13_, (3,0)) | [-1,1,-2] |
| 82 | Nc(2,0) | [0,-1,0] |
| 86 | Nc(2,1) | [1,-1,0] |
| 87 | Nc(2,3) | [0,-1,1] |
|  |  |  |
| 48 | Sac(P_23_) | [1,-1,-1] |
| 49 | Compl(P_23_) | [0,-1,-1] |
| 51 | sac_compl(P_23_, P_12_) | [0,-2,-1] |
| 53 | sac_compl(P_23_, P_13_) | [0,-1,-2] |
| 54 | Sac_nc(P_23_, (1,2)) | [0,0,-1] |
| 55 | Sac_nc(P_23_, (1,3)) | [0,-1,0] |
| 56 | Sac_nc(P_23_, (1,0)) | [0,-1,-1] |
| 57 | Sac_nc(P_23_, (2,1)) | [2,-2,-1] |
| 58 | Sac_nc(P_23_, (2,3)) | [1,-2,0] |
| 59 | Sac_nc(P_23_, (2,0)) | [1,-2,-1] |
| 60 | Sac_nc(P_23_, (3,1)) | [2,-1,-2] |
| 61 | Sac_nc(P_23_, (3,2) | [1,0,-2] |
| 62 | Sac_nc(P_23_, (3,0)) | [1,-1,-2] |
| 83 | Nc(3,0) | [0,0,-1] |
| 88 | Nc(3,1) | [1,0,-1] |
| 89 | Nc(3,2) | [0,1,-1] |
|  |  |  |
| 71 | Sac_sac(P_12_) | [-2,-2,0] |
| 72 | Sac_sac(P_13_) | [-2,0,-2] |
| 73 | Sac_sac(P_23_) | [0,-2,-2] |
| 74 | Sac_i_nc(P_i_,(1,2)) | [-2,0,-1] |
| 75 | Sac_i_nc(P_i_,(1,3)) | [-2,-1,0] |
| 76 | Sac_i_nc(P_i_,(2,1)) | [0,-2,-1] |
| 77 | Sac_i_nc(P_i_,(2,3)) | [-1,-2,0] |
| 78 | Sac_i_nc(P_i_,(3,1)) | [0,-1,-2] |
| 79 | Sac_i_nc(P_i_,(3,2)) | [-1,0,-2] |
| 80 | Sac_i(P_i_) | [-1,-1,-1] |

List of rules to test when the i-th lmer has excess score, each has 42 rules.

| 1^st^ lmer | 1,2,3,4,5,6,7,8,9,10,11,12,13,14,15,24,25,27,28,29,30,31,32,  33,34,35,36,37,38,71,72,73,74,75,76,77,78,79,80,81,84,85 |
| --- | --- |
| 2^nd^ lmer | 1,2,3,4,5,6,7,8,9,10,11,12,13,14,15,48,49,51,53,54,55,56,57,  58,59,60,61,62,28,71,72,73,74,75,76,77,78,79,80,82,86,87 |
| 3^rd^ lmer | 24,25,3,27,28,29,30,31,32,33,34,35,36,37,38,48,49,5,51,53,  54,55,56,57,58,59,60,61,62,71,72,73,74,75,76,77,78,79,80,83,88,89 |

Parallelization Configuration

Inside the python script, run_iTriplet.py, there is a line to define the available nodes. It looks like this:

nodes = ["compute-0-0.local", "compute-0-1.local"]

Change the name of nodes in your Linux cluster environment and specific –P option when running run_iTriplet.py.

Note that parallel and autonomous mode cannot be selected at the same time in current version. It has nothing to do with the iTriplet algorithm. The main reason is the extra development effort in post-processing. Since our focus in this paper is to present the research idea of iTriplet algorithm instead of producing a commercial product, therefore we will defer the enhancement to the near future.

Help text of iTriplet

An –h option is provided by run_iTriplet.py. It will print the following help text when specified:

| Usage: run_iTriplet.py with the following options  -i <fasta file> : input sequence file in fasta format  -l <integer> : anticipated size of motif, from 6 to 40. It is ignored if -A is specified  -d <integer> : maximum number of mutations allowed with respect to the motif, It is ignored if -A is  specified  -o <output> : file to store the output  Optional parameters:  -s <fraction> : anticipated percentage of sequences with the motif, default=1.0  -L <int-int> : range of motif length e.g. 6-20  -M : highest number of motifs to find, if more is found, program will abort. Default=1, recommend  10 in autonomous mode  -B : consider both strand if specified, default only given strand  -D <directory> : working directory  -P : run in parallel mode  -f <integer> : starting position, default is 1  -t <integer> : ending position, default to the end of sequence  -A : autonomous mode is on, default is off. iTriplet will explore various <l,d> models on behalf  of the user  When autonomous mode is on, parameters -l and -d are ignored  -h : print out this help text |
| --- |
